# The Risk of Colonoscopy‐related Bleeding in Patients With or Without Continued Treatment With an Antithrombotic Agent

**DOI:** 10.1002/deo2.70175

**Published:** 2025-07-17

**Authors:** Emi Nonaka, Ichitaro Horiuchi, Akira Horiuchi, Satoshi Ukai, Noriko Takahata, Kimihiko Oishi

**Affiliations:** ^1^ Department of Pediatrics The Jikei University School of Medicine Tokyo Japan; ^2^ Digestive Disease Center Showa Inan General Hospital Nagano Prefecture Japan; ^3^ Department of Pediatrics Jikei University Katsushika Medical Center Tokyo Japan

**Keywords:** antithrombotic agent | cold snare polypectomy | colonoscopy | EMR | endoscopic mucosal resection

## Abstract

**Objectives:**

We prospectively investigated the risk of colonoscopy‐related bleeding in relation to antithrombotic treatment.

**Methods:**

This prospective, observational, single‐center cohort study (NCT02594813) enrolled consecutive patients who underwent colonoscopy, including the removal of colorectal polyps, regardless of the continuation of antithrombotic treatment. The primary outcome measure was delayed bleeding in the patients who underwent a hot snare polypectomy and/or endoscopic mucosal resection in addition to a cold snare polypectomy (CSP) and required endoscopic treatment ≤2 weeks after the procedure. Secondary outcomes were immediate bleeding and the number of hemostatic clips used during the procedure.

**Results:**

From January 2019 to December 2023 at our institution, 1562 (mean age 75 years) and 15,769 (mean age, 64 years) patients underwent colonoscopy with or without antithrombotic treatment, respectively. Immediate bleeding following the removal of colorectal polyps, regardless of the polypectomy technique, occurred in 173 (33.86%) of the 511 patients with antithrombotic treatment, which was significantly more frequent than in 439 (9.44%) of the 4651 patients without antithrombotic treatment (*p* < 0.001). On the other hand, there was no significant difference in delayed bleeding after CSP between the two groups (0.41% vs. 0.11%, *p* = 0.15). However, the incidence of delayed bleeding following polypectomy was significantly higher in patients receiving antithrombotic treatment compared to those without it (seven of 511 [1.37%] vs. 12 of 4651 [0.26%], *p* = 0.0016).

**Conclusions:**

The risk of delayed bleeding after colonoscopy with removal of colorectal polyps was low despite continuation of antithrombotic therapy. www.clinicaltrials.gov (NCT02594813).

## 1 Introduction

The numbers of elderly individuals and patients being treated with an antithrombotic agent for the prevention of thrombosis due to cardiovascular or cerebrovascular disease is increasing worldwide [1]. Based on current practice guidelines in Japan and the U.S., a colonoscopy with the removal of colorectal polyps, including cold snare polypectomy (CSP), hot snare polypectomy, or endoscopic mucosal resection (EMR) is classified as a procedure that poses a high risk of bleeding, although screening colonoscopy including mucosal biopsy procedures has been shown to have a low risk of bleeding [2, 3]. A temporary cessation of treatment with antithrombotic agents is thus recommended. In contrast, the risk of bleeding after a colorectal polypectomy, which ranges from 0.3% to 10% depending on the polyp size, morphology, and resection technique (cold forceps polypectomy, CSP, hot snare polypectomy, or EMR) does not appear to be as serious when the risk of a thromboembolism from the temporary interruption of antithrombotic treatment is considered [4].

In July 2015, the ethics committee of Showa Inan General Hospital approved the use of colonoscopy, including CSP, for patients with small colorectal polyps while their treatment with antithrombotic agents is continued. This policy is described on the hospital's website (http://www.sihp.jp). One of our research group's subsequent studies demonstrated that a CSP of colorectal polyps <10 mm may not increase the risk of delayed bleeding while antithrombotic treatment is continued [5]. The use of colonoscopy for the removal of large colorectal polyps without the withdrawal of antithrombotic agents has continued at our hospital since January 2018. We conducted the present study to prospectively evaluate the risk of colonoscopy‐related bleeding during colonoscopy with CSP, hot snare polypectomy, and/or EMR in patients with or without antithrombotic treatment.

## 2 Patients and Methods

### 2.1 Study Design

This was a prospective, observational cohort study carried out at Showa Inan General Hospital, Japan. It adhered to the principles of the Declaration of Helsinki and received approval from the hospital's ethics committee. Written informed consent was obtained from all participants when their procedure was scheduled. The study has been registered with www.clinicaltrials.gov (NCT02594813).

### 2.2 Study Population

Patients who were referred to our hospital and scheduled for colonoscopy between January 2019 and December 2023 were enrolled. The inclusion criteria were patients who had undergone screening, diagnostic, or surveillance colonoscopy regardless of the continuation of antithrombotic treatment. Antithrombotic treatment is administered to lower the risk of thromboembolic events in patients with conditions such as atrial fibrillation, acute coronary syndrome, deep vein thrombosis, and cerebrovascular disorders. The exclusion criteria were American Society of Anesthesiologists class 3 or higher; poor bowel preparation (Boston Bowel Preparation Scale <6 points); endoscopic submucosal dissection, colorectal cancer or malignancy, inflammatory bowel disease, familial polyposis; emergent colonoscopy (hemodynamic instability and/or prolonged active gastrointestinal bleeding and/or requiring intensive care); known bleeding disorders or bleeding tendency, severe cardiopulmonary dysfunction, liver cirrhosis, severe chronic kidney disease (CKD) (estimated glomerular filtration rate <30 mL/min/1.73 m^2^), other malignancy, or severe infectious disease; and history of colorectal resection. Patients whose treatment with an antithrombotic agent was discontinued at their discretion were also excluded.

All patients had platelet counts >100,000/µL prior to the procedure. For patients on warfarin, prothrombin time and international normalized ratio (PT‐INR) was managed to be within the therapeutic range (2.0–3.0) on the day of colonoscopy, and those with supratherapeutic INR were rescheduled.

### 2.3 Procedures

All subjects underwent colonoscopy under propofol sedation by nurses supervised by endoscopists (Nichi‐Iko Pharmaceutical, Toyama, Japan) [6]. The colonoscopy pretreatment was performed using a polyethylene glycol solution (EA Pharmaceutical, Tokyo) [7]. All colonoscopy procedures were performed by one of the hospital's 10 experienced endoscopists, who each perform >500 endoscopies/year. Each colonoscopy was performed with the patient in the lateral decubitus position. Propofol was given by a bolus injection with an age‐adjusted standard protocol. The size of the videoscope used for the colonoscopies (PCF‐H290ZI; Olympus, Tokyo) had an 11.8‐mm insertion diameter, a 3.2‐mm accessory channel diameter, and a working length of 1330 mm.

All colorectal polyps, with the exception of tiny hyperplastic polyps in the rectum and distal sigmoid colon, were removed using CSP, hot snare polypectomy, and/or EMR. At our institution, polyps less than 10 mm in diameter are generally treated with CSP. Polyps measuring 10 mm or greater are considered for hot snare polypectomy or EMR. All of the CSPs were performed using dedicated cold snares with a 9‐mm maximum snare dia. (Exacto Cold Snare; US Endoscopy, Mentor, OH). The snare‐wire dia. of the dedicated cold snare is 0.30 mm. The snare used for the hot snare polypectomies and EMRs was a dual‐loop‐wire snare with a loop size of 33 mm or 16 mm (SN‐3316LX; Medico's Hirata, Osaka, Japan). An ERBE ICC 200 electrosurgical unit (Amco, Tokyo) was used in the Endocut mode with the effect 3 current set at the output limit 120 W and the forced coagulation current set at the output limit 35 W for the hot snare polypectomies and EMRs.

In the EMR procedure, a submucosal injection was made with a needle using a solution of epinephrine diluted 1:10,000 in saline; this injection was not used in the CSPs or hot snare polypectomies. For each polyp, 2–5 mL of solution was injected, and after the resection, the completeness of the polyp resection was checked endoscopically with the use of narrow band imaging and confirmed pathologically. If residual polyps were observed, they were resected again. Small polyps that were resected were collected in the suction channel. Larger polyps were collected using retrieval forceps without the use of the endoscopic suction channel, in order to avoid fragmenting the sample.

Prophylactic clipping after polyp removal was not routinely performed, regardless of polyp size or type of polypectomy. However, hemostatic clipping was performed for immediate bleeding or at the discretion of the endoscopist.

### 2.4 Data Collection

Monitoring and adverse events were prospectively recorded by the registered nurses. The indications for antithrombotic agents used, the snare used, the size, location, shape, and pathology of all polyps, and the number of hemostatic clips used were also prospectively recorded. The procedure duration was defined as the time from the initial insertion of the endoscope to its withdrawal. If patients had symptoms of abdominal pain, hematemesis, melena, or bloody stool after the colonoscopy, they were instructed to call the hospital and visit. After consultation with their physician, a complete blood count was performed if necessary. Additionally, patients with a hemoglobin decrease of ≥2 g/dL on the complete blood count underwent endoscopy for a search for bleeding stigmata. All patients who underwent colonoscopy, including a CSP, hot snare polypectomy, and/or EMR, either visited our hospital or were contacted by phone 2 weeks after the procedures to be informed of the pathological results. Adverse events and all gastrointestinal symptoms were recorded within 2 weeks after the endoscopic resections were performed.

### 2.5 Outcome Measures

The primary outcome measure of this study was delayed bleeding, which we defined as bleeding that required endoscopic treatment within 2 weeks after the patient's CSP, hot snare polypectomy, and/or EMR. The secondary outcome measures were immediate bleeding, defined as spurting or oozing that persisted for >30 s [8], and the total number of hemostatic clips used during the procedure.

### 2.6 Statistical Analyses

The results of our analyses are presented as means with standard deviations. For comparisons of categorical data, the χ^2^‐test with Yates' continuity correction was used when appropriate. When the sample sizes were small, Fisher's exact test was employed. For parametric data, Student's t‐test was used to compare two means. The factors associated with procedure‐related bleeding in patients on antithrombotic treatment were evaluated by both univariate and multivariate analyses. Variables with a *p*‐value <0.05 in the univariate analysis were included in the multivariate logistic regression model. The statistical analyses were conducted using GraphPad Prism Ver. 10.1.1 (GraphPad Software, San Diego, CA).

## 3 Results

**FIGURE 1:**
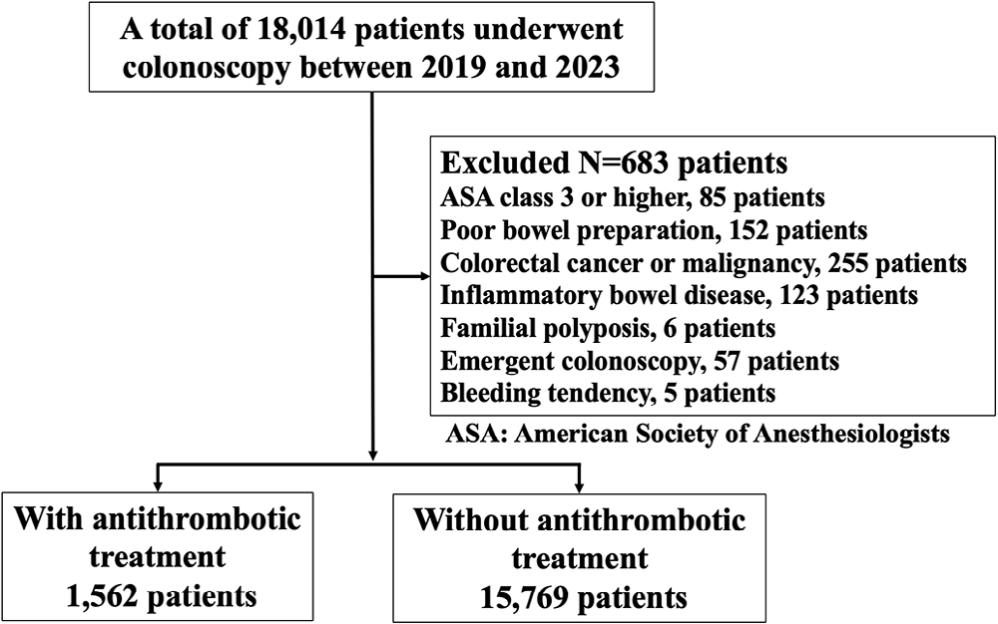
Study flow diagram of enrolled patients.

From January 2019 to December 2023, 1562 patients (mean age, 75 years) and 15,769 patients (mean age, 64 years) with and without antithrombotic treatment, respectively, underwent colonoscopy at our hospital and were finally enrolled in this study (Figure 1). The age of patients with antithrombotic treatment was significantly higher than that of the patients without antithrombotic treatment (*p* < 0.01) (Table 1). There were no significant differences in gender or the indications for colonoscopy between these groups, and no significant between‐group differences in the adenoma detection rate or the number of hot snare polypectomies or EMRs (Table 1). However, all of the following were significantly higher in the group under antithrombotic treatment compared to the group not being treated with antithrombotic agents: the average number of polyps/patient (0.52 vs. 0.30, *p* < 0.01), the number of CSPs (31.24% vs. 28.36%, *p* = 0.017), and the number of patients with hemostatic clips used (7.75% vs. 3.38%, *p* < 0.01). The characteristics of the size, location, shape, and pathology of the removed polyps did not differ significantly between the groups (Table 2).

**TABLE 1:** Clinical features of the patients who underwent colonoscopy with (+) or without (−) being treated with an antithrombotic agent (ATA).

	ATA (+)	ATA (−)	*p*‐Value
Colonoscopy, *n*	1562	15,769	
Age, years; mean [SD]	75 [16]	64 [15]	<0.01
Gender (female%)	34	36	0.88
Indications (%):			0.15
Hemo‐positive stool	65	55	
Screening	30	33	
Other	5	12	
Adenoma detection rate (%)	35	31	0.65
Mean no. of polyps/patient	0.52	0.30	<0.01
Cold snare polypectomy, *n* (%)	488 (31.24)	4473 (28.36)	0.017
Hot snare polypectomy or EMR, *n* (%)	39 (2.50)	478 (3.03)	0.27
Patient hemostatic clip used, *n* (%)	121 (7.75)	534 (3.38)	<0.01
Clips/patient clipped, median (range)	2 (1–5)	2 (1–3)	

Abbreviation: EMR: endoscopic mucosal resection.

**TABLE 2:** Comparison of colorectal polyps in the patients who underwent a cold snare polypectomy, hot snare polypectomy, or endoscopic mucosal resection (EMR), either with or without being treated with an antithrombotic agent (ATA).

	ATA (+), *n* (%)	ATA (−), *n* (%)	*p*‐Value
Total no. of polyps removed	811 (100)	4,777 (100)	
Characteristics of the removed polyps:			
Size, mm:			0.48
<5	299 (36.87)	1645 (34.44)	
6–10	434 (53.51)	2685 (56.21)	
11–19	51 (6.29)	277 (5.80)	
>20	27 (3.32)	170 (3.56)	
Location:			0.074
Left colon	423 (52.16)	2328 (48.73)	
Right colon	388 (47.84)	2449 (51.27)	
Shape:			0.23
Flat	153 (18.87)	882 (18.46)	
Sessile	598 (73.74)	3613 (75.63)	
Pedunculated	60 (7.40)	282 (5.90)	
Pathology:			0.50
High‐grade adenoma	5 (0.62)	39 (0.82)	
Adenoma	665 (82.00)	3821 (80.00)	
Sessile serrated adenoma/polyp	57 (7.02)	406 (8.50)	
Hyperplastic polyp	84 (10.36)	511 (10.70)	

Abbreviation: EMR: endoscopic mucosal resection.

The details of the patients who continued to receive antithrombotic treatment are shown in Table 3. The indications for antithrombotic treatment to reduce the risk of thromboembolic events were cerebrovascular disease (52.05%), atrial fibrillation (24.07%), acute coronary syndrome (11.14%), and deep vein thrombosis (10.56%). The antithrombotic agents used were aspirin (45.01%), warfarin (20.00%), clopidogrel (19.01%), warfarin + clopidogrel (6.02%), and direct oral anticoagulants (9.99%) (Table 3).

**TABLE 3:** The details of the 1562 patients who continued to be treated with an antithrombotic agent.

	Patients, *n* (%)
**Indications for antithrombotic therapy**	
Cerebrovascular disease	813 (52.05)
Atrial fibrillation	376 (24.07)
Acute coronary syndrome	174(11.14)
Deep vein thrombosis	165 (10.56)
Others	34 (2.18)
**Antithrombotic agents**:	
Aspirin	703 (45.01)
Warfarin	312 (20.00)
Chlopidogrel	297 (19.01)
Warfarin and chlopidogrel	94 (6.02)
Total direct oral anticoagulants:	156 (9.99)
Edoxaban	48 (3.07)
Apixaban	46 (3.07)
Dibgatran	32 (2.05)
Rivaroxoban	30 (2.05)

The data are *n* and (%).

As shown in Table 4, immediate bleeding following the removal of colorectal polyps, regardless of the polypectomy technique, occurred in 173 out of 511 (33.86%) patients among the 1562 colonoscopy patients who received antithrombotic treatment. This rate is significantly higher compared to the 1439 out of 4651 (9.44%) patients among the 15,769 colonoscopy patients who did not receive antithrombotic treatment (*p* < 0.001). On the other hand, there was no significant difference in delayed bleeding after CSP between the two groups (0.41% vs. 0.11%, *p* = 0.15). However, the incidence of delayed bleeding following polypectomy was significantly higher in patients receiving antithrombotic treatment compared to those without it (seven of 511 [1.37%] vs. 12 of 4651 [0.26%], *p* = 0.0016). Seven of the patients with delayed bleeding had been treated with warfarin + clopidogrel (*n* = 2), apixaban (*n* = 2), dabigatran (*n* = 2), or rivaroxaban (*n* = 1) (Table 5). Delayed bleeding was infrequent regardless of whether antithrombotic treatment was continued. None of the patients in this study experienced thromboembolic events after colonoscopy.

**TABLE 4:** The frequency of colonoscopy‐related bleeding in the patients treated with or without an antithrombotic agent (ATA).

Group	ATA (+)	ATA (‐)	*p*‐Value
Immediate bleeding after cold snare polypectomy Immediate bleeding after EMR	150/488 (30.73) 13/26 (50.00)	185/4473(4.14) 125/342 (36.55)	<0.001 0.17
Immediate bleeding after hot snare polypectomy or EMR	23/39 (58.97)	254/478 (53.14)	0.51
Immediate bleeding after the removal of colorectal polyps	173/511 (33.86) (95%CI: 29–37)	439/4,651 (9.44) (95%CI: 7.45–12.21)	<0.001
Delayed bleeding after cold snare polypectomy Delayed bleeding after EMR	2/488 (0.41) 3/26 (11.54)	5/4473 (0.11) 5/312 (1.60)	0.15 0.0013
Delayed bleeding after hot snare polypectomy or EMR polypectomy	5/39 (12.82)	7/478 (1.46)	0.0010
Delayed bleeding after the removal of colorectal polyps	7/511 (1.37) (95%CI: 1.12–1.73)	12/4651 (0.26) (95%CI: 0.21–0.33)	0.0016

The data are *n* (%). EMR: endoscopic mucosal resection.

**TABLE 5:** Details of the patients who had immediate bleeding or delayed bleeding, according to the type of antithrombotic agent.

	Immediate bleeding *n* (%)	Delayed bleeding *n* (%)
Total	173 (100)	7 (100)
Aspirin	59 (34.10)	0
Warfarin	28 (16.18)	0
Clopidogrel	24 (13.87)	0
Warfarin+clopidogrel	28 (16.18)	2 (28.57)
Edoxaban	7 (4.04)	0
Apixaban	9 (5.20)	2 (28.57)
Dibgatran	11(6.36)	2 (28.57)
Rivaroxoban	7 (4.05)	1 (14.29)

The multivariate analysis conducted after the univariate analysis revealed that hot snare polypectomy or EMR and large polyps (>20 mm) were significant factors associated with colonoscopy‐related bleeding in the patients on antithrombotic treatment (Table 6). Age, gender, the use of direct oral anticoagulants, the presence of cerebrovascular disease, prolonged procedure time, polyp location, and the presence of stalk were not significantly linked to bleeding related to colonoscopy in the patients on antithrombotic treatment.

**TABLE 6:** Univariate and multivariate analyses of factors associated with colonoscopy‐related bleeding in the patients treated with an antithrombotic agent.

	Univariate analysis		Multivariate analysis	
Variables	OR (95%CI)	*p*‐Value	OR (95%CI)	*p*‐Value
Age>70 years	1.21 (0.32–2.22)	0.56		
Gender male	1.33 (0.43–3.20)	0.43		
DOAC	1.63 (0.76–3.82)	0.11		
CVD	0.82 (0.23–2.13)	0.67		
CKD	7.70 (1.80–27.00)	0.003	2.24 (1.12–12.00)	0.54
CSP	4.50 (1.30–15.00)	<0.001	1.80 (0.56–2.30)	0.17
HSP or EMR	5.20 (1.50–22.00)	<0.001	4.40 (2.10–18.00)	<0.001
Prolonged procedure time >30 min	1.2 (0.34–2.40)	0.53		
Location, right colon	1.12 (0.55–3.24)	0.65		
The presence of stalk	1.53 (0.45–2.74)	0.37		
Large polyps >20 mm	12.00 (2.53–32.00)	<0.001	5.80 (2.20–12.00)	<0.001

Large polyps >20 mm 12.00 (2.53–32.00) <0.001 5.80 (2.20–12.00) <0.

Abbreviations: CI: confidence interval, CKD: chronic kidney disease, CSP: cold snare polypectomy, CVD: cerebrovascular disease, DOAC: direct oral anticoagulant, EMR: endoscopic mucosal resection, HSP: hot snare polypectomy, OR: odds ratio.

## 4 Discussion

In this study, we evaluated the risk of immediate or delayed bleeding following colonoscopic polypectomy in patients who continued or discontinued antithrombotic therapy. Our findings demonstrated that, while immediate bleeding was significantly more frequent in patients receiving antithrombotic therapy, delayed bleeding was overall rare. However, the incidence of delayed bleeding was significantly higher in the antithrombotic therapy group compared to the non‐antithrombotic group. Notably, CSP was associated with a low risk of delayed bleeding, regardless of whether antithrombotic therapy was continued, while hot snare polypectomy and EMR were associated with a higher risk.

Delayed bleeding is a critical concern in patients undergoing colonoscopic polypectomy, as it can lead to severe adverse events that may require endoscopic intervention. Our study highlights that while immediate bleeding is more common, particularly in patients on antithrombotic therapy, delayed bleeding, though rare, poses a more significant clinical risk. Fortunately, immediate bleeding can be managed during the procedure. Table 4 shows that delayed bleeding occurred more frequently in patients continuing antithrombotic therapy (1.37%) compared to those not on therapy (0.26%, *p* = 0.0016). This finding emphasizes the importance of careful post‐procedural monitoring in this patient population.

The risk of delayed bleeding varied based on the type of polypectomy performed. Specifically, CSP exhibited a low delayed bleeding rate, even among patients on antithrombotic therapy (0.41% vs. 0.11%, *p* = 0.15), suggesting that CSP is a safer approach for this group. Conversely, hot snare polypectomy and EMR were associated with a significantly higher risk of delayed bleeding in patients under antithrombotic therapy (12.82% vs. 1.46%, *p* = 0.0010). On the other hand, another study demonstrated that, even when anticoagulants were continued, the risk of delayed bleeding after a hot snare polypectomy or EMR did not increase compared to when anticoagulants were discontinued [9].

In our group's previous study, hot snare polypectomy that injures submucosal vessels was observed to pose a high bleeding risk, whereas a CSP that involves fewer submucosal vessels was observed to have a low bleeding risk in anticoagulated patients [10]. The delayed bleeding after a hot snare polypectomy or EMR is thought to occur due to vascular damage caused by tissue injury and necrosis from electrocoagulation. In a comparison of post‐polypectomy mucosal defects observed the day after hot snare polypectomy and the day after CSP, it was reported that the mucosal defects expanded after hot snare polypectomy, whereas they shrank after CSP [11]. Based on that report and our present results, we speculate that hot snare polypectomy and EMR with electrocautery cause more tissue damage compared to CSP. The use of electrocautery in these procedures likely contributes to increased tissue injury, delayed mucosal healing, and subsequent bleeding.

Our multivariate analysis identified hot snare polypectomy or EMR and large polyps (>20 mm) as significant risk factors for delayed bleeding in patients on antithrombotic therapy (Table 6). We had also observed that CKD was significantly associated with bleeding related to colonoscopic procedures in patients on direct oral anticoagulants (DOACs) [12]. Given these findings, careful patient selection and procedure choice are essential. While CSP can be safely performed without discontinuation of antithrombotic agents, hot snare polypectomy and EMR require more cautious decision‐making. For high‐risk patients, prophylactic measures such as clipping, careful anticoagulation management, and post‐procedural observation should be considered to minimize bleeding risk.

Many guidelines recommend that anticoagulation be resumed as soon as possible after 7 days of anticoagulant interruption in patients with a low risk of thrombosis, and that anticoagulation be resumed with heparin bridging within 3 days in patients at a high risk of thrombosis [13, 14, 15]. An analysis of 6264 patients with atrial fibrillation treated with rivaroxaban or apixaban indicated that rivaroxaban was associated with a higher risk of bleeding but a similar risk of stroke/thromboembolism and death compared to apixaban [16]. In a study of 381,054 patients, apixaban was associated with a lower risk of gastrointestinal bleeding compared to dabigatran and rivaroxaban [17]. The differences in bleeding risk among DOACs were not clarified in the present study.

There are several study limitations to address. It was conducted at a single domestic facility, and the numbers of patients and lesions were small. The choice of endoscopic treatment for colorectal polyps was based on the endoscopist's judgment. Although general indications for polypectomy are established, the decision was not strictly standardized, and it depended on the individual endoscopist's discretion. In addition, a few of the enrolled patients were being treated with DOACs. Although many colonoscopy patients at our hospital are administered DOACs, they were not enrolled in this study because they did not take the medication on the day of the colonoscopy.

Due to our study's small sample size, it was not possible to evaluate delayed bleeding after colonoscopic procedures according to the type of DOACs. Moreover, the proportion of immediate bleeding in our study population was high. The definition of immediate bleeding may vary across studies, and further investigation into the definition of immediate bleeding is necessary. Lastly, we did not assess the risk of delayed bleeding in patients taking anticoagulants by using the Endoscopic Resection Group of the Spanish Society of Endoscopy score [18].

In conclusion, the risk of delayed bleeding after colonoscopy with removal of colorectal polyps was low despite continuation of antithrombotic therapy. Our study is strengthened by its prospective design, large sample size, and comprehensive follow‐up, allowing for robust comparison of bleeding risks in patients with and without continued antithrombotic therapy. The findings provide clinically relevant evidence supporting the safety of CSP in patients on antithrombotic agents and highlight risk factors for delayed bleeding after polypectomy. In contrast, hot snare polypectomy and EMR pose a higher risk and require careful patient selection and peri‐procedural management. These findings support a tailored approach to colonoscopic polypectomy in anticoagulated patients to balance bleeding risk and thromboembolic prophylaxis. Future studies with larger cohorts and randomized designs are needed to refine guidelines for the management of antithrombotic therapy in endoscopic procedures.

## Ethics Statement

This study was approved by the hospital's ethics committee on October 21, 2018 (No. 2018‐03).

## Consent

All of the enrolled patients gave their written informed consent for their data to be published when the procedure was scheduled.

## Conflicts of Interest

The authors declare no conflicts of interest.

## Clinical Trial Registration

The study was registered at www.clinicaltrials.gov (NCT02594813).

## Data Availability

The data underlying this article will be shared on reasonable request to the corresponding author.
